# Hydrophobic Sand Is a Non-Toxic Method of Urine Collection, Appropriate for Urinary Metal Analysis in the Rat

**DOI:** 10.3390/toxics5040025

**Published:** 2017-10-11

**Authors:** Jessica F. Hoffman, Vernieda B. Vergara, Steven R. Mog, John F. Kalinich

**Affiliations:** 1 Internal Contamination and Metal Toxicity Program, Armed Forces Radiobiology Research Institute, Uniformed Services University, Bethesda, MD 20889, USA; vernieda.vergara.ctr@usuhs.edu (V.B.V.); John.Kalinich@usuhs.edu (J.F.K.); 2 Office of Food Additive Safety (OFAS), CFSAN/FDA/DHHS, College Park, MD 20740, USA; steven.mog@fda.hhs.gov

**Keywords:** urine, metal contamination, internal contamination, rodent

## Abstract

Hydrophobic sand is a relatively new method of urine collection in the rodent, comparable to the established method using a metabolic cage. Urine samples are often used in rodent research, especially for biomarkers of health changes after internal contamination from embedded metals, such as in a model of a military shrapnel wound. However, little research has been done on the potential interference of hydrophobic sand with urine metal concentrations either by contamination from the sand particulate, or adsorption of metals from the urine. We compare urine collected from rats using the metabolic cage method and the hydrophobic sand method for differences in metal concentration of common urinary metals, and examine physical properties of the sand material for potential sources of contamination. We found minimal risk of internal contamination of the rat by hydrophobic sand, and no interference of the sand with several common metals of interest (cobalt, strontium, copper, and manganese), although we advise caution in studies of aluminum in urine.

## 1. Introduction

The development of the full metal-jacketed bullet around the time of the Spanish-American War in 1898 improved survivability from battle wounds and increased the probability of embedded metal fragments in survivors [1]. Embedded metal fragments were initially considered inert, and a low health risk, until the appearance of several case reports on medical issues associated with embedded fragment wounds suffered during wartime many years prior to manifestation of the adverse health effect [2,3,4,5,6,7].

The majority of research into health effects of embedded metals has been conducted in the context of the safety of implanted devices [8], with little focus on long-term health effects of military-relevant metals and metal mixtures [9] until several U.S. military personnel were wounded by depleted uranium (DU) fragments during Operation Desert Storm in 1991. Standard medical protocol was to leave fragments in place for the life of the individual. However, due to DU’s chemical and radiological properties and little information available on the long-term health effects of embedded DU, the need for research into the biokinetics and toxicology of DU became clear. The Armed Forces Radiobiology Research Institute (AFRRI) developed and validated a rodent model system to assess the health effects of embedded metal fragments [10]. Results of this investigation led to a reassessment of the Department of Defense (DoD) fragment removal policy for DU, recommending excising fragments larger than 1 cm in diameter and patients be followed for any long-term adverse health effects [11].

The concern over DU embedded fragment health effects led to the search for replacement materials for DU munitions. Several tungsten-based compositions were then tested for adverse health effects using the AFRRI embedded fragment model system, but it was discovered that the tungsten/nickel/cobalt composition induced malignant, highly aggressive rhabdomyosarcomas at the implantation sites [12], while a tungsten/nickel/iron composition did not result in any tumor formation [13,14]. Underscoring our current lack of knowledge regarding long-term health effects of military-relevant metal fragments is the high number of military personnel returning wounded from the recent conflicts in Iraq and Afghanistan. Between multiple munition types, vehicle armor, and improvised explosive devices (IEDs), the list of metals and metal mixtures that may potentially be found as embedded fragments is extensive. As a result, the DoD and the Department of Veterans Affairs (DVA) have developed a list of “metals of concern” with respect to embedded fragments [15], but the biokinetic and toxicological properties of many of these metals when embedded as fragments are not yet known.

In an effort to address these problems, our ongoing research projects investigate biokinetic, toxicological, and carcinogenic effects of several military-relevant metals by using the implanted metal rodent system and examining changes in urine, serum, and tissue samples. The analysis of urinary metal levels is an excellent method by which to identify the composition of embedded metal fragments as well as a way to monitor metal solubilization from the fragment over time [16]. Rodent urine is commonly collected through the use of metabolic cages, which can be stressful for the animals [17,18] and requires habituation [19]. An alternate method of rodent urine collection, hydrophobic sand, has recently come to the market. Originally developed for urine collection in the cat, hydrophobic sand is a biodegradable material with a non-toxic hydrophobic coating that causes urine to pool on its surface, making it easy to collect. The material is currently available as “LabSand” to the scientific community, or “Kit4Cat” commercially. A review of both products’ safety data sheets [20,21], as well as telephone communication with the supplier (Coastline Global, Inc., Palo Alto, CA, USA), indicate they are identical. If urine is to be assayed for biomarkers and dissolved metals in our embedded metal fragment model system, it is imperative to know whether the hydrophobic sand could contaminate urine samples with extraneous metals or adsorb baseline metals from urine. Previously we compared metabolic cage and hydrophobic sand urine collection methods for stress and clinical markers and found no significant differences that would compromise normal urine markers [22]. Here, we used the same urine samples from that experimental set to determine if there is a difference in urine metal concentration between the two collection methods. Further, we thoroughly examined the physical properties of LabSand and Kit4Cat to discover if hydrophobic sand could adsorb metals from urine, or leech out any metals and contaminate urine samples through contact with urine before collection, or from being ingested by the rat.

## 2. Materials and Methods 

The animals, urine collection methods, experimental design, and urine samples are the same as those reported in Hoffman et al., 2017 [22]. These methods are repeated here in brief. All other methods described afterward are unique to this work. 

### 2.1. Animals

Male Sprague Dawley rats (Envigo, Frederick, MD, USA) were maintained on a 12:12 light:dark cycle with access to food and water *ad libitum*. Rats were pair-housed except during urine collection periods. Rats underwent no treatment or experimental conditions beyond exposure to both urine collection methods. All procedures involving animals were approved by the AFRRI Institutional Animal Care and Use Committee under protocol 2016-05-006.

### 2.2. Urine Collection Methods

#### 2.2.1. Metabolic Cages

Animals were in a standard circular metabolic cage with a wire mesh floor with urine collected in a Nalgene tube at the bottom of a funnel system. Urine could only be collected at the end of the session. 

#### 2.2.2. Hydrophobic Sand

Animals were in a rectangular microisolator cage with the sand lining the bottom of the cage in place of regular bedding; urine pools on top of the sand, which is then collected with a pipette. For each rat, we collected urine every half hour and was subsequently pooled at the end of the session. The pooled urine sample for each animal was used for analysis in the current report.

### 2.3. Experimental Design for Urine Collection

The experimental design and urine sample collection schedule is illustrated in Figure 1 of Hoffman et al., 2017 [22]. We used a within-subjects crossover design where rats were randomly assigned to two groups: (A) was the metabolic cage followed by LabSand, (B) was LabSand followed by the metabolic cage, *n* = 4 for each group for a total of 8 animals in both collection methods, serving as their own control. Both groups were run simultaneously under the same testing conditions. There was a total of 5 collection sessions (a 2 h, a 4 h, and three separate 6 h sessions), each separated by a rest period of at least 48 h. The method crossover occurred after the last session and the entire pattern was repeated. Food and water were not provided to any animal during urine collection sessions, but each animal was provided with a water replacement gel in a plastic cup (HydroGel^®^, Clear H_2_O, Westbrook, ME, USA). The gel material can be eaten by the rat for hydration but does not drip and dilute urine samples as a water bottle could.

### 2.4. Creatinine Concentration in Urine

Creatinine concentrations in urine collected during metabolic cage and LabSand sessions were reported in Table 1 of Hoffman et al., 2017 [22], and subsequently used to normalize metal concentrations reported here. Creatinine concentrations were also determined for urine collected from the bladder of all 8 rats after euthanasia using the same assay as before. Briefly, a colorimetric creatinine assay kit (Oxford Biomedical Research, Inc., Oxford, MI, USA) was used to determine the difference in absorbance wavelength after picric acid is added to urine, then again after addition of an acid reagent. Values were compared against a creatinine standard curve, all absorbance values were read on a spectrophotometer (SpectraMax 190, SoftMax Pro 2.0 software, Molecular Devices, Sunnyvale, CA, USA).

### 2.5. Examining Potential Internalization of Hydrophobic Sand by Rats

One month after completion of the metabolic cage versus LabSand experiment, rats were humanely euthanized by isoflurane exposure followed by exsanguination and confirmatory pneumothorax and lung and gut tissues collected to be examined for evidence of inhalation or ingestion of sand particulate. Of the 8 rats that had gone through the metabolic cage/LabSand crossover method experiment, 3 rats were placed in cages with LabSand 2 h prior to euthanasia (“Acute Exposure”), and the other 3 rats were left in their home cage for the same period before euthanasia (“Past Exposure”, equating to 1 month between last sand exposure and euthanasia). The stomach was opened and physically examined for any evidence of ingestion of hydrophobic sand. Additionally, lung tissue from 3 naïve rats (“No Exposure”) never exposed to hydrophobic sand were collected. 

All lung tissue was fixed in 10% buffered formalin, processed, and embedded in paraffin, sectioned in 5–6 µm thick slices onto glass slides, and stained with hematoxylin and eosin (HE) stain for histology by the AFRRI pathology division. Slides were then examined by a board-certified veterinary pathologist using a BX51 Olympus microscope at 100× magnification under both bright light and simple polarizing light using a U-ANT analyzer and a U-POT polarizer. Representative photomicrographs were chosen to demonstrate an arteriole, terminal bronchiole, and a larger bronchiole to show the internal positive control for birefringence (normal supportive fibrous connective tissue-collagen around the vessel and major airway).

### 2.6. Assessment of Cytotoxicity

#### 2.6.1. Cell Line and Media

V79 Chinese hamster lung fibroblasts were purchased from the American Type Culture Collection (ATCC, Manassas, VA, USA) and maintained in Dulbecco’s Modified Eagle’s Medium (D-MEM, Invitrogen, Grand Island, NY, USA) with 10% fetal bovine serum (FBS, Invitrogen) at 37 °C in a humidified atmosphere of 5% CO_2_ in air. Cells were passed twice per week and were used between passages 5 and 12.

#### 2.6.2. Cell Treatment

LabSand (1 g) was mixed in 10 mL of D-MEM with 10% FBS for 24 h at room temperature by gentle mixing on a nutator. The mixture was then centrifuged at 400× *g* for 10 min at room temperature and the supernatant filtered through a 0.2 µm to create an extraction solution. The extraction solution was then tested at full strength (undiluted, “100% Extract”), diluted 1:10 with D-MEM with 10% FBS (“10% Extract”), or diluted 1:100 with D-MEM with 10% FBS (“1% Extract”). Cells were plated on 96-well tissue culture plates at a predetermined concentration for maximum response in a toxicity assay (described below). In replicates of 6, cells were incubated for 24 h in the following groups: Control (D-MEM with 10% FBS media only), 1% Extract, 10% Extract, or 100% Extract. After the 24 h incubation cells were assayed for viability.

#### 2.6.3. Viability Assay

Metabolic viability (MTT assay) was assessed using the CellTiter 96^®^ Aqueous One Solution Cell Proliferation Assay kit (Promega Corporation, Madison, WI, USA). The assay for metabolic viability is based upon the ability of dehydrogenase enzyme systems, located in the cell mitochondria, to reduce a tetrazolium compound to a colored formazan product, which is easily detected colorimetrically. Briefly, one hour prior to the termination of the 24 h treatment incubation period, 10 µL of CellTiter 96^®^ Aqueous One Solution Reagent was added to each well of the plate and the plate returned to the incubator for 1 h. After this time, the absorbance was determined at 490 nm using a microplate reader (SpectraMax Model 250 Microplate Spectrophotometer, Molecular Devices Corporation, Sunnyvale, CA, USA). Metabolic viability of the extract-treated cells was normalized to the media-only control cells.

### 2.7. Analyses of Physical Properties of LabSand

#### 2.7.1. Determination of Hydrophobic Sand Particle Size

5 g of LabSand or Kit4Cat brands of hydrophobic sand was placed in a Scienceware Mini-sieve Micro Sieve Set (Bel-Art Products, Wayne, NJ, USA) using the following mesh screen sizes: 25, 35, 45, and 60 standard mesh (0.71, 0.50, 0.35, and 0.25 mm, respectively). The apparatus was gently shaken by hand for 5 min, then each of the various fractions were weighed and normalized to the total weight of the recovered fractions in 3 separate replications. 

#### 2.7.2. Imaging of Hydrophobic Sand Particles

A small sample of LabSand was placed on a slide and examined at 2× under bright light on an Olympus BX61 microscope using an Olympus DP72 camera (Olympus America, Inc., Center Valley, PA, USA).

### 2.8. Metal Analysis by ICP-MS

All compounds used in this study were obtained from Sigma-Aldrich Co. (St. Louis, MO, USA) or Thermo Fisher Scientific (Pittsburgh, PA, USA) and were of the highest grade available. Plastic ware and other disposables were also obtained from Thermo Fisher Scientific.

Samples were first analyzed using survey scans across the full atomic mass range of likely metal analytes via ICP-MS. Metals observed at higher amounts than the control were identified for further quantification, as were analytes that displayed larger-than-expected peaks. ICP-MS operating conditions and parameters can be found in Supplementary Materials Table S1. Limit of Detection (LoD)/Limit of Quantitation (LoQ), in ppb, are as follows: Al—0.38/0.44; Co—0.03/0.06; Cu—0.24/0.54; Pb—0.02/0.04; Sr—0.01/0.05; Zn—2.80/3.01; Fe—1.08/1.85.

#### 2.8.1. Urine Metal Analysis

Urine samples from metabolic cage, LabSand, and bladder collections described above were diluted in 2% nitric acid and measured by ICP-MS. Samples were then normalized against creatinine (mg/mL) to give a ng metal/mg creatinine value.

#### 2.8.2. Analysis of Metal Recovery from Hydrophobic Sand

In order to assess whether metals in a sample could nonspecifically bind to LabSand, solutions of various metals including Al, Co, Cu, Pb, Sr, and Zn were mixed with LabSand (0.1 g) for various times. Samples were centrifuged (13,000× *g* for 10 min at room temperature) and the resulting supernatant removed and analyzed for metal content using ICP-MS. Recovery of metals in contact with LabSand for 5, 15, or 60 min were compared to control.

#### 2.8.3. Digestion of Hydrophobic Sand by Synthetic Rat Gut Fluid and Nitric Acid

Approximately 0.1 g of LabSand was treated with 1.0 mL of simulated gastric fluid by mixing on a nutator for 2 h at room temperature. Simulated gastric fluid was prepared according to Ansoborlo et al. (1999) [23]. The extraction mixture was centrifuged at 13,000× *g* for 10 min at room temperature and the resulting supernatant removed and analyzed for metal content using ICP-MS in survey scan mode followed by quantitation of those metals present. Similarly, at 13,000× *g* for 10 min at room temperature and the resulting supernatant removed and analyzed for metal a 70% nitric acid (Optima Grade, Fisher Scientific, Pittsburgh, PA, USA) solution was used to determine maximum metal that could be digested out of the hydrophobic sand.

#### 2.8.4. Metal Analysis of HydroGel^®^

To determine metal content in the HydroGel^®^ hydration cup gel samples, approximately 0.1 g of gel were cut from the HydroGel^®^ and placed in tared glass vials and mass determined. Nitric acid (5 mL of 70% Optima Grade) was added and the gel allowed to dissolve overnight at room temperature. Aliquots of the dissolved gel were analyzed for metal content via ICP-MS in survey scan mode followed by quantitation of those metals present.

#### 2.8.5. Treatment of Metabolic Cage Pieces with Simulated Urine

Components of the metabolic cage apparatus that were in extended contact with urine during the collection procedure were assessed for removable copper contamination using the following procedure. Simulated urine solution was prepared following the method of Issacson (1968) [24]. Metabolic cage pieces (collection ring, funnel, collection cylinder) were washed with a laboratory detergent (Contrex, DeCon Labs, King of Prussia, PA, USA) and rinsed extensively with tap water. One group of metabolic cage components were allowed to air dry, while the second set was further washed with deionized water (18 mΩ, Elga Purelab Water System, Highwycombe, Bucks, UK) before air drying. The metabolic cage components were then treated with the simulated urine solution. The collection ring and collection tubes were filled with 5 mL of simulated urine solution for 2 h at room temperature. The simulated urine fluid was then collected. For the metabolic cage funnel, 5 mL of simulated urine solution was passed through the funnel 5 times before collecting. Aliquots of the collected simulated urine solution were analyzed for copper content using ICP-MS.

### 2.9. Statistical Analysis

For cell cytotoxicity, the percent change from control for each extract was compared to 100% (not toxic) using a one sample *t*-test. Extracts were then compared to each other using a one-way ANOVA. Animal urine metal concentrations were analyzed as a within-subjects two-tailed *t*-test comparison between collection methods for each session time. Urine collected from the bladder at the time of animal euthanasia was used as a control for urinary metal concentration in a one-way ANOVA within-subjects comparison with the third 6 h session metabolic cage and LabSand urine samples. Metals after gastric solution or nitric solution digestion were compared via *t*-test. Particle size, aluminum, and strontium distribution by fractions were each analyzed by two-way ANOVA, followed by a post-hoc Sikak’s multiple comparisons test if there was a main effect between fractions. Changes in metal concentration from a spiked metal standard mixed with LabSand for 5, 15, or 60 min were analyzed by subtracting the 0 time metal concentration from each time point and compared to 0 PPB (no change) using a one-sample *t*-test. All analyses used GraphPad Prism Software (version 7.01, La Jolla, CA, USA). *P* values less than 0.05 were considered significant.

## 3. Results

### 3.1. Risk of Internalization and Cytotoxicity

**Figure 1:**
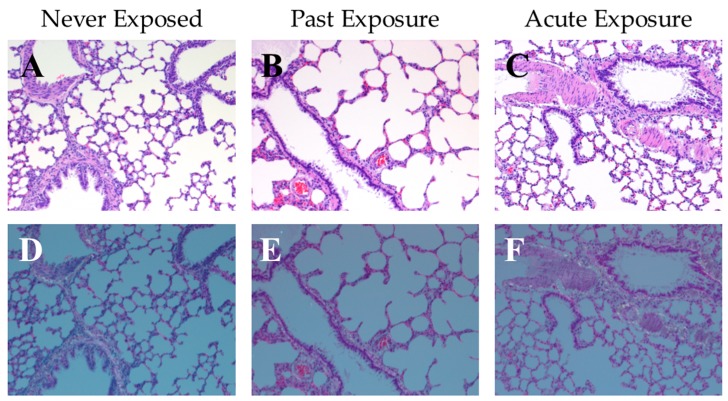
Representative photos of rat lung tissue at 100×; sections show an inflated area that included an arteriole, terminal bronchiole, and a larger bronchiole. (**A**–**C**) Hematoxylin and eosin (HE) stained tissue under bright light microscopy, and (**D**–**F**) corresponding HE stained tissue under polarized light. (**A**,**D**) tissue from a naïve rat that were never exposed to hydrophobic sand (“Never Exposed”); (**B**,**E**) tissue from a rat that underwent all LabSand experimental sessions, and was euthanized 1 month after the conclusion of the experiment with no further exposure to hydrophobic sand (“Past Exposure”); (**C**,**F**) tissue from a rat that underwent all LabSand experimental but was also placed in a cage with hydrophobic sand for 2 h prior to euthanasia (“Acute Exposure”).

If hydrophobic sand is to be used as a method of urine collection in rodents, it is important to determine whether the animals exposed to hydrophobic sand are at risk of internalization either through inhalation of small particles or ingestion of the material, posing a potential health risk and/or source of contamination of subsequent urine and tissue samples. One month after the conclusion of the metabolic cage vs. LabSand experiment in Hoffman et al., 2017 [22], all 8 rats were euthanized and their stomach contents examined for presence of any sand grain particles. Three rats were placed in a cage with hydrophobic sand for 2 h prior to euthanasia. No grains of hydrophobic sand were found in any stomach contents. Additionally, lung tissue was collected from 9 total animals: 3 naïve control rats that never underwent the metabolic cage vs. LabSand experiment and thus were never exposed to hydrophobic sand (deemed the “Never Exposed” group), 3 rats that were part of the experiment but were not reintroduced to hydrophobic sand prior to euthanasia (the “Past Exposure” group), and 3 rats that were part of the experiment but also reintroduced to the hydrophobic sand for 2 h prior to euthanasia (the “Acute Exposure” group). HE stained lung tissues from all 9 rats were examined microscopically under both bright light and polarized light, which would highlight any significant silica particulate foreign material trapped in the tissue. All lung tissues were normal, with no silicate crystals identified and no significant aggregates of any inflammatory cells around terminal airways in any group (Figure 1A–F).

**Figure 2:**
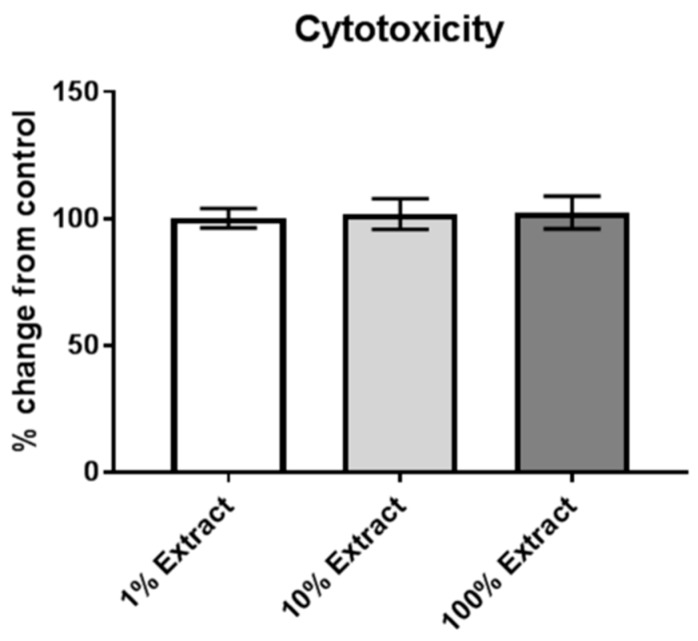
Metabolic viability (MTT) assay of V79 Chinese hamster lung fibroblast cells exposed to varying concentrations of media that has been mixed with hydrophobic sand.

Next, we wanted to determine if anything toxic to cells could leech off hydrophobic sand and pose a health risk if a rat was to ingest or inhale particulate. LabSand was agitated gently in cell media on a nutator for 24 h, then the media filtered of all particulate to create an “extract.” V79 Chinese hamster lung fibroblast cells were plated onto 96-well plates and treated with normal media or a dilution of the filtered media mixed with LabSand (“1% Extract” is a 1:100 dilution, “10% Extract” is a 1:10 dilution, and “100% Extract” is undiluted extract media). After 24 h of exposure to the various concentrations of LabSand-exposed media, cells were evaluated for survival using a metabolic viability (MTT) assay and calculated as percent change from the normal media control where 100% indicates no change from control. The media extracts are not significantly different from each other (one-way ANOVA, F_2,15_ = 0.262, *p* = 0.77), nor is any dilution’s percent change from control significantly different from 100% (one-sample *t*-tests: 1% Extract, t_5_ = 0.089, *p* = 0.93; 10% Extract, t_5_ = 0.712, *p* = 0.51; 100% Extract, t_5_ = 0.906, *p* = 0.41) (Figure 2).

### 3.2. LabSand vs. Metabolic Cage: Metal in Urine

Past research into the effects of an embedded fragment model on metal concentrations in urine has used metabolic cages for rodent urine collection. Hoffman et al., 2017 [22] suggests hydrophobic sand is a useful alternative urine collection method, but we need to ensure there is no metal contamination of urine samples from the hydrophobic sand before moving forward with this collection method. To do this, we scanned the urine samples from the metabolic cage versus LabSand experiment, as well as urine collected from the bladder at euthanasia, for cobalt (Co), copper (Cu), strontium (Sr), aluminum (Al), manganese (Mn), zinc (Zn), lead (Pb), and uranium (U) using ICP-MS and normalized to creatinine for each sample. Zn, Pb, and U concentrations were below the detectible limit, but the rest of the metals were compared within each session time using a within-subjects *t*-test (metabolic cage vs. LabSand collection method). Urine collected from the bladder was never in contact with hydrophobic sand or the metabolic cage equipment, so this was used as a control for urinary metal concentration in a one-way ANOVA within-subjects comparison with the third 6 h session metabolic cage and LabSand urine samples.

**Figure 3:**
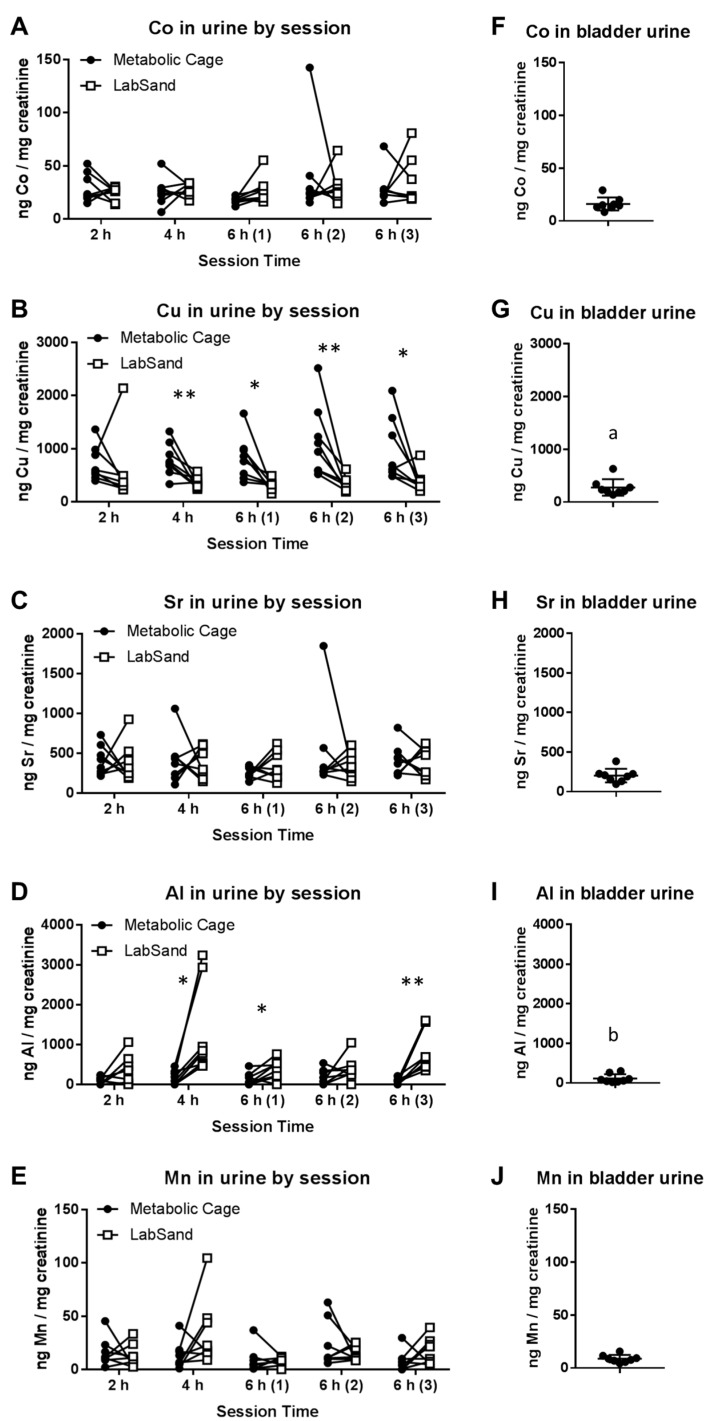
Metal concentrations in urine comparing metabolic cage and LabSand collection methods (**A**–**E**) and urine collected from the bladder (**F**–**J**). Graphs (**F**–**J**) were kept at the same *Y*-axis value as the corresponding session graph (i.e., A-F, B-G, C-H, D-I, E-J) because bladder urine metal concentrations are directly compared to the 6 h-3 session. Asterisks indicate significant differences in urine metal concentration between collection methods for that session (* *p* < 0.05, ** *p* < 0.01), (a) denotes a significant difference in metal concentration between bladder urine and metabolic cage urine from session 6 h-3, and (b) denotes a significant difference in metal concentration between bladder urine and LabSand urine from session 6 h-3.

For cobalt, there were no significant differences in urine concentration between metabolic cage and LabSand urine collection methods in any collection session (2 h session, t_7_ = 0.922, *p* = 0.39; 4 h session, t_7_ = 0.311, *p* = 0.76; 6 h-1, t_7_ = 2.251, *p* = 0.06; 6 h-2, t_7_ = 0.576, *p* = 0.58; 6 h-3, t_7_ = 0.516, *p* = 0.62) (Figure 3A). There were also no significant differences between bladder urine or the 6 h metabolic cage or LabSand urines (F_(4.5,10.3)_ = 2.864, *p* = 0.112) (Figure 3F data compared with Figure 3A, 6 h-3 session data). For all time points except the 2 h session, copper in urine from the LabSand collection method is lower than copper in urine from the metabolic cage method (2 h session, t_7_ = 0.630, *p* = 0.55; 4 h session, t_7_ = 3.505, ** *p* < 0.01; 6 h-1, t_7_ = 3.235, * *p* < 0.05; 6 h-2, t_7_ = 3.513, ** *p* < 0.01; 6 h-3, t_7_ = 2.435, * *p* < 0.05) (Figure 3B). This would suggest LabSand is somehow absorbing copper out of the urine that pools on it. However, when bladder urine concentrations were compared to the third 6 h session methods (F_(1.2,8.4)_ = 7.534, * *p* < 0.05), we found that copper in the LabSand urine is not significantly different from copper in the bladder urine (Tukey’s multiple comparison test, *p* = 0.530), but rather copper in the metabolic cage urine is surprisingly higher than copper in the bladder urine (Tukey’s, ^a^ *p* < 0.05) (Figure 3G data compared with Figure 3G, 6 h-3 session data).

For strontium, there were no significant differences in urine concentration between metabolic cage and LabSand urine collection methods in any collection session (2 h session, t_7_ = 0.209, *p* = 0.84; 4 h session, t_7_ = 0.152, *p* = 0.88; 6 h-1, t_7_ = 1.223, *p* = 0.26; 6 h-2, t_7_ = 0.775, *p* = 0.46; 6 h-3, t_7_ = 0.280, *p* = 0.79) (Figure 3C). There were also no significant differences between bladder urine or the 6 h metabolic cage or LabSand urines (F_(1.7,11.9)_ = 3.79, *p* = 0.06) (Figure 3H data compared to Figure 3C, 6 h-3 session data). Aluminum is significantly higher in urine from the LabSand collection method compared with the metabolic cage collection method in the 4 h (t_7_ = 3.105, * *p* < 0.05), first 6 h (t_7_ = 2.88, *p* < 0.05), and third 6 h (t_7_ = 3.952, ** *p* < 0.01) sessions, but not the 2 h (t_7_ = 1.925, *p* = 0.096) or second 6 h (t_7_ = 1.561, *p* = 0.162) sessions (Figure 3D). When bladder urine concentrations were compared to the third 6 h session methods (F_(1.0,7.2)_ = 14.06, ** *p* < 0.01), we found that aluminum in the metabolic cage urine is not significantly different from aluminum in the bladder urine (Tukey’s multiple comparison test, *p* = 0.355), but is higher in the LabSand urine than in the bladder urine (Tukey’s, ^b^ *p* < 0.05) (Figure 3I data compared to Figure 3D, 6 h-3 session data).

For manganese, there were no significant differences in urine concentration between metabolic cage and LabSand urine collection methods in any collection session (2 h session, t_7_ = 0.492, *p* = 0.638; 4 h session, t_7_ = 1.724, *p* = 0.128; 6 h-1, t_7_ = 0.332, *p* = 0.75; 6 h-2, t_7_ = 1.01, *p* = 0.346; 6 h-3, t_7_ = 1.497, *p* = 0.178) (Figure 3E). There were also no significant differences between bladder urine or the 6 h metabolic cage or LabSand urines (F_(1.5,10.6)_ = 2.082, *p* = 0.177) (Figure 3J data compared to Figure 3E, 6 h-3 session data).

### 3.3. Potential Sources of Contamination

**Figure 4:**
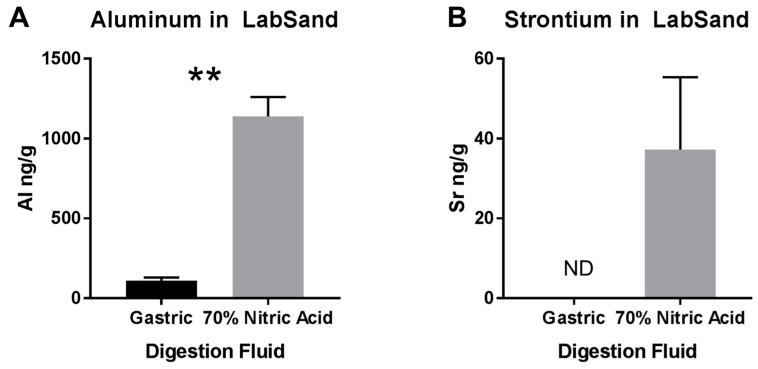
Digestion of LabSand brand hydrophobic sand in either synthetic gastric fluid or 70% nitric acid. (**A**) Aluminum and (**B**) Strontium extracted from LabSand under the two digestive conditions. ** *p* < 0.01, ND = not detected.

Significantly higher levels of aluminum in urine from the LabSand urine samples compared to bladder samples suggests aluminum may be leeching out of the hydrophobic sand to contaminate urine, but significantly higher levels of copper in urine from the metabolic cage urine samples compared to bladder samples without any difference between LabSand and bladder urine concentrations suggest metal contamination of urine is not necessarily straightforward contamination by LabSand. To investigate potential sources of metal contamination in urine samples, we conducted several further tests with LabSand and metals. First, we wanted to determine what metals were present, and in what concentrations, in LabSand brand hydrophobic sand under maximum digestive conditions, and what concentrations may be extracted out of that sand if they were to be ingested by a rat. Approximately 0.1 g samples of LabSand were mixed with either a synthetic gastric fluid solution or 70% nitric acid solution (for maximum metal extraction) for 2 h, which is the normal transit time through the rat stomach [25]. Samples were filtered and measured for metal via ICP-MS. In the survey scan, only aluminum and strontium appeared in any quantity above control, and were subsequently quantitated. Significantly less aluminum (mean: 111.1, SD: 33.79) was able to be extracted from the LabSand digested in synthetic gastric fluid than from LabSand digested in 70% nitric acid (mean: 1140, SD: 208.8 ng/g sand; t_4_ = 8.42, ** *p* < 0.01) (Figure 4A), which represents the maximum amount of aluminum that could be extracted from the LabSand. Strontium levels after extraction from LabSand digested with synthetic gastric fluid were below the limits of detection, and levels were very low when digested with the maximum condition, 70% nitric acid mean: 37.23, SD: 31.40 ng/g sand) (Figure 4B).

**Figure 5:**
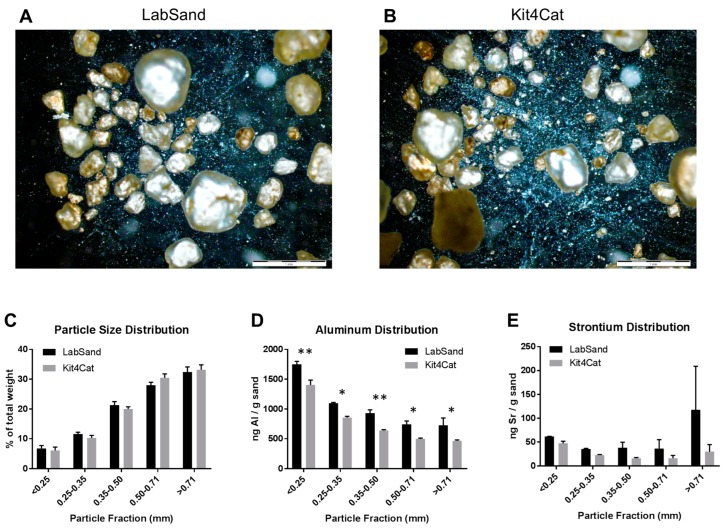
Distribution of particles in hydrophobic sand brands. Brightfield images under 2× magnification for (**A**) LabSand and (**B**) Kit4Cat sand samples; (**C**) Particle size distribution across 5 size fractions for both brands of hydrophobic sand; (**D**) Aluminum distribution across size fractions for both brands; (**E**) Strontium distribution across size fractions for both brands. * *p* < 0.05, ** *p* < 0.01.

Next, we wanted to know the particle size distribution of hydrophobic sand, as well as any potential differences in metal concentrations within that particle size distribution. We examined both LabSand and Kit4Cat brands, which the manufacturer lists as nearly identical. Visual inspection of both hydrophobic sand brands under brightfield at 2× magnification reveal a similar distribution of particles of varying sizes, from large pebble-like structures to fine dust (Figure 5A,B). A sieve system was then used to separate LabSand or Kit4Cat particulate into 5 fractions according to size and each fraction was calculated as a percent of the total weight, then compared as distribution across fractions within a brand as well as between brands within fraction by two-way ANOVA. There was a significant main effect of particle fraction distribution (F_4,20_ = 187.6, *p* < 0.0001) for both brands, but no difference between brands in the distribution across fractions (F_1,20_ < 0.001, *p* = 0.99) and no interaction effect (F_4,20_ = 0.99, *p* = 0.44) (Figure 5C). In both brands of hydrophobic sand, more than 50% of the particles were larger than 0.50 mm (LabSand, 60.3%; Kit4Cat, 63.7%) and only a small percentage is smaller than 0.25 mm (LabSand, 6.8%; Kit4Cat, 6.1%).

Fractions were then subjected to the same extraction process for metals using the 70% nitric acid solution as before and analyzed for aluminum and strontium by ICP-MS, as those were the metals above control on the survey scan for the whole sample for each brand. There was a main effect of fraction (F_4,20_ = 100.9, *p* < 0.0001) where aluminum concentration was distributed unequally between particle size fractions. For both brands, the highest concentration of aluminum occurred in the smallest fraction size (<0.25 mm; LabSand mean: 1747.66, SD: 91.05 ng/g sand; Kit4Cat mean 1402.36, SD: 144.74 ng/g sand). There was also a main effect of brand (F_1,20_ = 59.7, *p* < 0.0001) but no interaction effect (F_4,20_ = 0.297, *p* = 0.877). The concentration of aluminum was significantly greater in LabSand than in Kit4Cat within every particle size fraction: <25 mm (t_20_ = 4.323, *p* < 0.01), 0.25–0.35 mm (t_20_ = 2.98, *p* < 0.05), 0.35–0.50 mm (t_20_ = 3.634, *p* < 0.01), 0.50–0.71 mm (t_20_ = 3.086, *p* < 0.05), >0.71 mm (t_20_ = 3.254, *p* < 0.05) (Figure 5D). LabSand and Kit4Cat had no significant differences in strontium concentration or distribution across particle size fractions. There was no significant main effect of particle fraction (F_4,20_ = 0.989, *p* = 0.436), brand (F_1,20_ = 2.705, *p* = 0.116), or interaction (F_4,20_ = 0.552, *p* = 0.700) (Figure 5E). The concentration of strontium in the less than 25 mm particle fraction is 61.65 (SD: 0.560) ng/g LabSand and 47.308 (SD: 7.742) ng/g Kit4Cat.

Now that we knew aluminum and strontium were the only metals at risk of leeching out of LabSand into the animals if ingested, or potentially into the urine as contamination, the next question was whether metals in the urine might bind to the sand and be pulled from urine before it could be collected. Urine was collected every half hour, so the longest time any pool of urine was in contact with the hydrophobic sand was 30 min. We used a spiked standard for each metal of interest (Al, Sr, Cu, Co, Sr, Zn) in water and mixed (not placed on top, to replicate a worst-case scenario) with 0.1 g of LabSand for 5, 15, or 60 min, then filtered, measured for metal concentration, and the spiked control value was subtracted from each time point to give the change in metal concentration. These values were then compared to 0 PPB by a one-sample *t*-test to determine if there was a significant change from the spiked metal concentration (Table 1). We used water instead of a synthetic urine solution because proteins in synthetic urine could interact with the metals and obscure any interaction between metals and LabSand. Aluminum had no significant change in concentration from spiked control at either the 5 min (t_2_ = 3.372, *p* = 0.078) or 60 min (t_2_ = 2.742, *p* = 0.111) time points, but showed a small but statistically significant increase of 0.654 ng/mL at the 15 min time point (t_2_ = 17.61, ** *p* < 0.01). Strontium had a small but statistically significant increase in concentration from spiked control at all three time points: 1.143 ng/mL at 5 min (t_2_ = 10.33, ** *p* < 0.01), 0.810 ng/mL at 15 min (t_2_ = 6.372, * *p* < 0.05), and 1.090 ng/mL at 60 min (t_2_ = 11.68, ** *p* < 0.01). Copper had a very small but statistically significant increase in concentration over spiked control (0.057 ng/mL) only at the 60 min time point (t_2_ = 109.3, ** p < 0.01). Cobalt had no significant change from control at any time point (5 min, t_2_ = 0.161, *p* = 0.887; 15 min, t_2_ = 1.089, *p* = 0.390; 60 min, t_2_ = 0.046, *p* = 0.968).

Other potential sources of contamination may exist beyond the hydrophobic sand itself. One such source of metal contamination could be the HydroGel^®^ cups. We cut out 3 samples each from 3 different gels and analyzed them for metals via ICP-MS. Only aluminum appeared in the survey scan and subsequently quantified. We found a mean concentration of 2463 (SD 2486) ng Al/g hydrogel (Min: 28.9 ng/g, Median: 1200 ng/g, Max: 7058 ng/g). Additionally, since copper was higher in urine from the metabolic cage collections than either the LabSand or the bladder urine, which suggests something else is adding copper to the urine, we suspected the tap water used to rinse the metabolic cages after cleaning may be leaving residue after air-drying, so we tested all of the lab sinks and our 18 Ω water supply for metal. Results are shown in Table 2. Sink 1 was the source of water used for washing the metabolic cages, though all four sink locations were surprisingly high in both copper and strontium concentrations compared to the 18 Ω water supply.

To follow up our discovery of high metals in the sink tap water, we ran a short experiment with the collection apparatus of the metabolic cage where we washed the collection pieces (collection ring, funnel, collection cylinder) with Contrex and allowed to air dry as was done in the original experiment, or we included an additional rinse step of deionized water between the wash with Contrex and tap water and air drying , then used a synthetic urine solution to simulate a collection period of 2 h before measuring for copper by ICP-MS. Copper concentrations (mean, SD) are presented as PPB (ng/mL) in Table 3. The additional rinse with deionized water did not reduce copper deposits from the initial wash (two-way ANOVA, F_1,12_ = 0.7551, *p* = 0.402), though the artificial urine picked up lower levels of copper levels from the funnel than from either the ring or collection container (F_2,12_ = 7.292, *p* < 0.01), suggesting the longer real urine would sit in those pieces during a collection session, the more likely they are to pick up small copper deposits from the plastic. If metabolic cages are to be used in future experiments, the method of washing the pieces must be carefully considered.

## 4. Discussion

Recently we have shown a relatively new method of rodent urine collection using hydrophobic sand (brand names LabSand or Kit4Cat) to be as efficient as using the metabolic cage collection method for short-term volume collections with no significant changes to clinically-relevant urinary markers or properties [22]. In order to use the hydrophobic sand collection method for future work examining urine samples in a rodent model of metal shrapnel wounds, we also wanted to ensure that the period of exposure to hydrophobic sand did not present an ingestion or inhalation risk, alter natural background metal concentrations in urine, nor contaminate urine samples with extraneous metals from the sand. To accomplish this, we examined urine samples from the Hoffman et al., 2017 [22] study for changes in baseline urine metal concentrations between the metabolic cage and LabSand collection methods, as well as compared collection samples to urine collected from the bladder after euthanasia. Additionally, the stomach contents of all animals were examined for evidence of ingestion of sand, and lungs examined for evidence of inhalation of sand. We found no evidence of sand in the stomach contents of any of the 8 rats in our study, including three rats that were placed in LabSand for a two hour period directly prior to euthanasia. Similarly, a study using Kit4Cat hydrophobic sand found only 2 grains of sand in the stomach of 1 out of 10 mice [26], suggesting rodents do not typically ingest the sand material. Further, we found no evidence of sand particulate in the lung tissue of the rats in our study whether they were exposed to LabSand during only the collection periods or exposed to an additional two hours of sand directly prior to euthanasia. Comparing the lung tissue to naïve rats that were never exposed to hydrophobic sand at all, we also conclude from the histopathology that there was no inflammatory response or tissue damage from any exposure to the sand. If, however, sand was accidently ingested or inhaled by a rodent, we also determined the sand would pose a minimal risk of toxicity to tissue because there was no effect of increasing exposures of LabSand in media on the metabolic viability of Chinese hamster lung fibroblast cell cultures.

Next, by comparing background metal concentrations in urine collected using both the metabolic cage and hydrophobic sand methods, we found no significant differences between the method of collection for cobalt, strontium, or manganese concentration. Copper concentrations were lower in urine collected using the hydrophobic sand method than metabolic cage for 4 out of 5 session times. We thought this was due to adsorption of copper into the LabSand material, but comparing both methods’ urine samples to urine collected from the bladder, which never had direct contact with either the metabolic cage apparatus or LabSand material, we found that copper is in fact higher in urine collected from the metabolic cage samples, and copper concentrations are not different between LabSand and the bladder samples. Metabolic cage parts are made of Nalgene, and do not contain intrinsic copper in the material. In examining the sources of water used to wash the cage parts, however, we discovered high copper concentrations in the tap water compared to purified water, which suggests droplets dried after washing deposited small amounts of copper onto the sides of the collection materials that were then picked up in the urine as it flowed down into the collection cup. To further confirm lack of contamination of copper concentrations from the hydrophobic sand, we showed that copper was not found in LabSand material after digestion by nitric acid or an artificial gastric juice solution, and there was no change in copper concentration from a spiked standard after 5, 15, or 60 min of mixing with LabSand material.

Aluminum was the only other metal that had any significant difference in urine concentration between the metabolic cage and LabSand collection methods, with it being higher in LabSand urine samples in 3 out of 5 collection sessions. Comparison with bladder urine concentration revealed that aluminum was, in fact, higher in the LabSand collection samples, with no difference from the metabolic cage collected samples. Nitric acid digestion of LabSand material revealed high concentrations of aluminum in the sand particulate, although internal contamination of the rat is of minimal risk—artificial gastric juice pulled significantly less aluminum out of the LabSand material after digestion. Aluminum had the highest concentration in the smallest particle fraction of both brands of hydrophobic sand, potentially posing a source of internal contamination if inhaled, but since the smallest particle fraction also makes up the lowest percentage of fraction sizes and lack of evidence of sand particulate in the lung, increased concentration of aluminum in urine from internalization of hydrophobic sand is highly unlikely, and thus could come from contact with the sand material itself. A 15 min period of mixing an aluminum spiked solution with LabSand did result in a significant increase of 0.654 ng/mL, but this is nowhere near enough to account for the difference of several hundred to several thousand ng/mL of aluminum in urine from the LabSand collection method over the metabolic cage collection method, especially since urine was collected from the LabSand surface every half hour. One other potential source of aluminum was the HydroGel^®^ water replacement material provided to each rat. We found very high levels of aluminum in some of the gel samples, which could have contaminated the urine either through direct contact, though that was rare, or ingestion of the gel material increased urine concentration temporarily. However, it is difficult to make that determination because we did not measure intake of gel for each animal, and animals in the metabolic cages did have access to gel cups from the same lot as the animals in LabSand collection sessions, and would require further, more precise study to determine the true level of contamination risk.

We conclude that the use of hydrophobic sand is an acceptable alternative method to the traditional metabolic cage method for urine collection in the rodent, especially for short-term (6 h or less) collection periods when total urine recovery is not necessary. For most metals of interest we examined (cobalt, strontium, copper, and manganese), hydrophobic sand has no effect on background natural urinary metals, nor does it appear to adsorb or otherwise contaminate metal concentration in urine. Aluminum urine concentration, however, may be confounded by the use of hydrophobic sand, and analyses of aluminum in urine should be done with caution. However, we believe the contamination risk would be greatly minimized by immediate collection of urine pools from the hydrophobic sand surface and not using the HydroGel^®^ material as a source of water replacement, as it has high concentrations of aluminum itself.

## Figures and Tables

**Table 1:** Changes in spiked metal concentrations after various times mixing with LabSand.

Metal	Time Spent Mixing with LabSand		
	5 min	15 min	60 min
Aluminum	1.254 (*0.644*)	0.654 (*0.064*) **	2.35 (*1.484*)
Strontium	1.143 (*0.192*) **	0.810 (*0.220*) *	1.090 (*0.162*) **
Copper	−0.013 (*0.14*)	0.074 (*0.117*)	0.057 (*0.001*) **
Cobalt	0.204 (*0.136*)	0.100 (*0.180*)	−0.006 (*0.240*)
Zinc	−2.917 (*0.340*) **	−2.717 (*0.251*) **	−2.26 (*0.265*) **
Lead	0.205 (*0.015*) **	0.270 (*0.022*) **	0.189 (*0.036*) *

Values presented as mean (*SD*), with units in PPB (ng/mL). A positive mean indicates metal concentration increased over spiked standard after exposure to LabSand, while a negative mean indicates metal concentration decreased from spiked standard after exposure to LabSand. * *p* < 0.05, ** *p* < 0.01.

**Table 2:** Metal concentrations in lab sink water.

Source	Metal Concentration, PPB (ng/mL)				
	Co	Cu	Sr	Al	Mn
18 Ω	−0.020	0.065	0.190	0.560	−0.010
Sink 1	0.095	413.150	270.950	5.610	3.380
Sink 2	0.180	1411.000	238.700	5.950	3.170
Sink 3	0.100	303.950	258.400	3.635	7.780
Sink 4	0.315	105.550	179.200	0.995	4.225

**Table 3:** Copper in artificial urine after sitting on metabolic cage collection parts.

Treatment	Copper Concentration, PPB (ng/mL)		
	Ring	Funnel	Container
Millipore Rinse	0.616 (*0.459*)	0.153 (*0.050*)	0.934 (*0.538*)
Tap Water alone	0.520 (*0.211*)	0.100 (*0.061*)	0.694 (*0.218*)

Values presented as mean (*SD*).

## Supplementary Materials

The following are available online at www.mdpi.com/2305-6304/5/4/25/s1, Table S1: ICP-MS operating conditions and parameters.

## Abbreviations

DU	Depleted uranium
AFRRI	Armed Forces Radiobiology Research Institute
LS	LabSand
MC	Metabolic cage
ICP-MS	Inductively coupled plasma mass spectroscopy
