# Supplementary material for: Hydrophobic Sand Is a Non-Toxic Method of Urine Collection, Appropriate for Urinary Metal Analysis in the Rat
Source: Toxics. 2017 Oct 11;5(4):25. doi: 10.3390/toxics5040025 (PMC5750553; doi:10.3390/toxics5040025)
Supplement: Supplementary file 1 [file toxics-05-00025-s001.pdf]

Supplementary Materials: Hydrophobic sand is a non-toxic method of urine collection, appropriate for urinary metal analysis in the rat

Jessica F. Hoffman, Vernieda B. Vergara, Steven R. Mog and John F. Kalinich

Table S1. ICP-MS operating conditions and parameters.

**ICP-MS operating conditions and parameters**	
Instrument Parameters	
Nebulizer type	Concentric
Spray chamber	Conical, with impact bead
Sampler cone	Nickel, 1mm orifice diameter
Skimmer cone	Nickel, 0.7 mm orifice diameter
Sample uptake rate	1.0 ml/min
Sample read delay	45 sec
Plasma conditions	
RF power	1400 W
Plasma argon gas flow	13.0 L/min
Auxiliary argon gas flow	0.80 L/min
Nebulizer gas flow	0.91 L/min
Mass spectrometer settings	
Scanning mode	Peak jump
Sweeps	100
Dwell time	500 µs
Channels/mass	1
Acquisition time	10 sec
Number of readings/replicate	3
Number of replicates	2
